# High gene flow maintains genetic diversity following selection for high EPSPS copy number in the weed kochia (Amaranthaceae)

**DOI:** 10.1038/s41598-020-75345-6

**Published:** 2020-11-02

**Authors:** Sara L. Martin, Leshawn Benedict, Wei Wei, Connie A. Sauder, Hugh J. Beckie, Linda M. Hall

**Affiliations:** 1 Agriculture and Agri-Food Canada, Ottawa Research and Development Centre, 960 Carling Avenue, Ottawa, ON Canada; 2 State Key Laboratory of Vegetation and Environmental Change, Institute of Botany, Chinese Academy of Sciences, Beijing, China; 3 Australian Herbicide Resistance Initiative, School of Agriculture and Environment, University of Western Australia, Perth, Australia; 4 Agricultural Food and Nutritional Science, University of Alberta, Edmonton, AB Canada

**Keywords:** Ecology, Genetics, Evolution, Evolutionary genetics, Molecular evolution, Population genetics, Agroecology, Plant molecular biology, Plant stress responses

## Abstract

Kochia, a major weed species, has evolved resistance to four herbicide modes of action. Herbicide resistance appears to spread quickly, which could result in diminished standing genetic variation, reducing the ability of populations to adapt further. Here we used double digest restriction enzyme associated sequencing to determine the level of gene flow among kochia populations and whether selection for glyphosate resistance reduces genetic variation. Canadian Prairie populations show little to no genetic differentiation (F_ST_ = 0.01) and no correlation between genetic and geographic distance (r^2^ = − 0.02 p = 0.56), indicating high gene flow and no population structure. There is some evidence that kochia populations are genetically depauperate compared to other weed species, but genetic diversity did not differ between glyphosate susceptible and resistant populations or individuals. The inbreeding coefficients suggest there are 23% fewer heterozygotes in these populations than expected with random mating, and no variation was found within the chloroplast. These results indicate that any alleles for herbicide resistance can be expected to spread quickly through kochia populations, but there is little evidence this spread will reduce the species’ genetic variation or limit the species’ ability to respond to further selection pressure.

## Introduction

A key area of interest in evolutionary biology is understanding the consequences of selection for genetic diversity and the future ability of populations to adapt. One potential consequence of strong selection is a reduction in the raw material, the genetic variation, available for selection^1^. At the individual level, this reduction can be detected in persistent genomic signatures of selective sweeps such as those associated with human evolution (reviewed by^2,3^), animal e.g.^4,5^ and crop domestication e.g.^6,7^. The evolution of herbicide resistance (HR) in agricultural weeds results from strong selection pressure. The spread of these resistance alleles through populations provides a growing number of study systems for understanding the consequences of selective sweeps at the individual and population level^8–10^. For practical reasons, weed managers are interested in understanding whether or not the standing variation for other herbicide modes of action is likely to be lost from populations following this selection. This could delay the evolution of additional HR within the population resulting from the time expected for new mutations conferring this resistance to arise in the population^9,11–14^. However, even when selection pressure is strong, loss in genetic variation depend on multiple factors, including the genetic basis of the resistance, mating system, population size, spatial or temporal variation in selection pressure, and gene flow^9^.

Kochia (*Bassia scoparia* (L.) A.J. Scott syn. *Kochia scoparia* (L.) Schrad.) is native to Europe and Asia and introduced to Canada, the United States, Africa, and South America. It is an annual noted for early germination, tolerance of arid and saline conditions, and a tumble weed habit. Kochia is wind pollinated and produces a large amount of pollen. However, it is also self-compatible, and as a result, the species is likely predominately outcrossing, but with high levels of variablity^15^. It has North American herbarium collections dating from the 1880s (reviewed by^15^). Kochia was found to be the fastest spreading alien species in the western USA from 1880 to 1980^16^. It causes significant yield losses (30–60%) in crops such as winter wheat and sugar beet^15^, a problem exacerbated by the evolution of multiple herbicide resistance^17^.

The species has evolved resistance to four herbicide modes of action^18^. This includes resistance to photosystem II inhibitors^18^, acetolactate synthase (ALS) inhibitors^19,20^, synthetic auxins^21^ and glyphosate^22–24^. Individuals with multiple herbicide resistance to all four modes of action have been detected in Kansas^17^, while individuals combining ALS, glyphosate (GR) and synthetic auxin resistance were documented in Alberta, Canada in 2017^25^. Kochia populations with ALS inhibitor resistance were first detected in Canada in 1988^26^ and the point mutations conferring this resistance have become nearly ubiquitous throughout the Prairie provinces in less than 20 years^27^. Glyphosate resistance was first detected in Kansas in 2007^22^ and was widespread in the USA’s Great Plains and confirmed in all three Canadian Prairie provinces by 2013^28–30^. This suggests that glyphosate resistance arose de novo in Texas after 33 years of glyphosate use^31^ and 11 years of intensified glyphosate use following the introduction of glyphosate resistant crops^31,32^. This resistance appears to have then spread through populations, rather than being a common variant in the species’ standing variation or emerging repeatedly de novo across the range. The glyphosate resistance (GR) mechanism described for kochia is increased copy number and expression of the 5-enolpyruvylshikimate-3-phosphate synthase (EPSPS) enzyme, which is inhibited by glyphosate in susceptible plants^33^. EPSPS copy number correlates with glyphosate resistance, with four or more copies resulting in resistance^17,24^. Inheritance of increased copy number follows a single locus Mendelian pattern, as the gene copies are in a tandem array on a single chromosome^34^. At this time, no other mechanisms of glyphosate resistance for kochia have been described.

While the spread of herbicide resistance genes, via pollen and seed, depends on the interconnectivity of populations, the selection of novel herbicide resistance from standing variation in a population depends on variation being available. In addition to selection for multiple HRs, often which have resulted from changes at a single locus (ALS, EPSPS, and photosystem II inhibitors), kochia’s history as an introduced species may limit available variation as only a subset of the variation available within a species is expected to be introduced to a new region^35–37^. Here we used double digested restriction enzyme associated markers and 26 populations from the Canadian Prairies to understand the current genetic variation in kochia populations and how selection for glyphosate resistance may have changed this variation. Specifically, we investigated three questions: (1) what is the level of gene flow and inbreeding among the kochia populations? (2) what is the current level of genetic diversity in these populations? and (3) do populations where high EPSPS copy number (EPSPSCN) has been introduced or individuals with high EPSPSCN show evidence of reduced diversity?

## Results

No individual sampled from a susceptible population had increased EPSPSCN relative to ALS, while resistant populations were generally a mixture of individuals with and without increased EPSPSCN. In one population, identified as 10% resistant in the initial screening of 100 individuals^29^, none of the 12 individuals sampled had increased EPSPSCN (Table 1), as a result, this pair was excluded from comparisons of resistant and susceptible populations.

**Table 1:** Number of individuals with fewer (n_S_) or more (n_R_) than 4 copies of EPSPS relative to ALS included in estimates of: allelic richness (A_R_); observed heterozygosity (H_O_), expected heterozygosity (H_E_), bootstrapped estimate of inbreeding coefficient (F_IS_); the average proportion of polymorphic nucleotide sites within individuals by 10^−3^ (P_n_), the average proportion of loci that showed variability within individuals (H_L_), population-specific estimates of genetic differentiation (F_ST_ ) (BayeScan), and geographic distance (km) between population pairs.

	Susceptible populations								Resistant populations									Distance between paired sites (km)
	n_S_	A_R_	H_O_	H_E_	P_n_	H_L_	F_IS_	F_ST_	n_S_	n_R_	A_R_	H_O_	H_E_	P_n_	H_L_	F_IS_	F_ST_	
AB-01	12	1.27	0.18	0.28	2.43	0.11	0.36	0.02	2	7	1.26	0.21	0.27	2.59	0.13	0.28	0.01	2.76
AB-02	12	1.27	0.20	0.28	2.56	0.12	0.32	0.02	4	8	1.26	0.19	0.26	2.57	0.15	0.29	0.03	2.85
AB-03	12	1.26	0.18	0.26	2.45	0.12	0.33	0.02	4	4	1.26	0.17	0.28	2.21	0.14	0.42	0.01	4.95
AB-04	11	1.26	0.23	0.26	2.83	0.14	0.16	0.03	5	7	1.27	0.22	0.27	2.87	0.14	0.23	0.01	2.19
MB-01	12	1.26	0.22	0.26	2.74	0.13	0.16	0.04	6	6	1.27	0.17	0.28	2.28	0.11	0.25	0.02	1.04
MB-02	10	1.27	0.24	0.28	2.95	0.14	0.23	0.02	4	5	1.26	0.23	0.27	2.80	0.13	0.12	0.02	28.68
SK-01	12	1.27	0.23	0.26	2.96	0.14	0.17	0.02	0	12	1.27	0.22	0.27	2.75	0.17	0.18	0.01	10.28
SK-02	12	1.27	0.22	0.27	2.87	0.14	0.28	0.02	0	10	1.26	0.25	0.27	3.20	0.13	0.18	0.02	4.77
SK-03	12	1.27	0.23	0.27	3.01	0.14	0.06	0.01	6	6	1.26	0.23	0.27	2.88	0.14	0.39	0.02	2.03
SK-04	12	1.26	0.20	0.27	2.63	0.12	0.20	0.01	4	6	1.26	0.24	0.28	2.89	0.14	0.28	0.02	6.16
SK-05	12	1.27	0.28	0.27	3.44	0.17	0.17	0.02	0	12	1.26	0.16	0.26	2.33	0.11	0.01*	0.06	26.99
SK-06	12	1.26	0.22	0.26	2.90	0.14	0.25	0.03	6	6	1.27	0.21	0.27	2.67	0.13	0.4	0.03	3.3
SK-07	12	1.26	0.23	0.26	2.95	0.14	0.20	0.03	12	0**	1.26	0.27	0.26	3.51	0.17	0.19	0.02	34.46

*Bootstrapped confidence interval included zero.

**The initial survey screen of population (n = 100) indicated 10% resistant individuals^29^, but no individuals sampled here had high EPSPSCN and both populations from the pair were coded as susceptible in population level comparisons.

In total, after following the STACKS pipelines^38^ for SNP discovery and genotyping, 360 (94%) and 362 (95%) of individuals had sufficient coverage in the de novo and reference based pipelines, respectively. However, the sibling sets were excluded in the majority of analyses, resulting in the inclusion of 89 high EPSPS individuals and 206 low EPSPS individuals (Table 1). For the reference based pipeline, the dataset included 3248 variable (polymorphic) loci with 10.6% missing data, while the de novo pipeline had 3173 variable loci with 11.2% missing data. Overall, 1.29% of nucleotides examined were polymorphic across alleles and nucleotide diversity (π) was 0.0036. Most consensus reference loci (99.6% of 6041 polymorphic and fixed loci) and 83.9% (of the 5626 polymorphic and fixed) of the de novo loci mapped to the genome (Bowtie2^39^). In total, 3440 loci were identified by both pipelines. Population analyses were run on all four data sets, but statistical results were very similar (Supplementary Table S2) and only the reference based pipeline’s results are presented. The minor allele frequency (MAF) in the reference-based set of loci averaged 0.19.

### Population structure and gene flow

**Figure 1:**
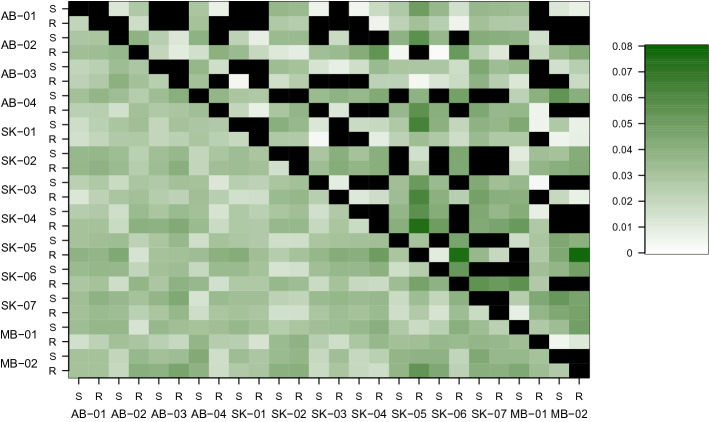
Between population heat map with higher values as more intensely coloured with Nei’s genetic distance (D_ST_; ranged from 0.01 to 0.05) below the diagonal and F_ST_ values (ranged from 0 to 0.08) above the diagonal. Values that were not considered statistically different from 0 (bootstrap p-value < 0.05) are coded in black.

**Figure 2:**
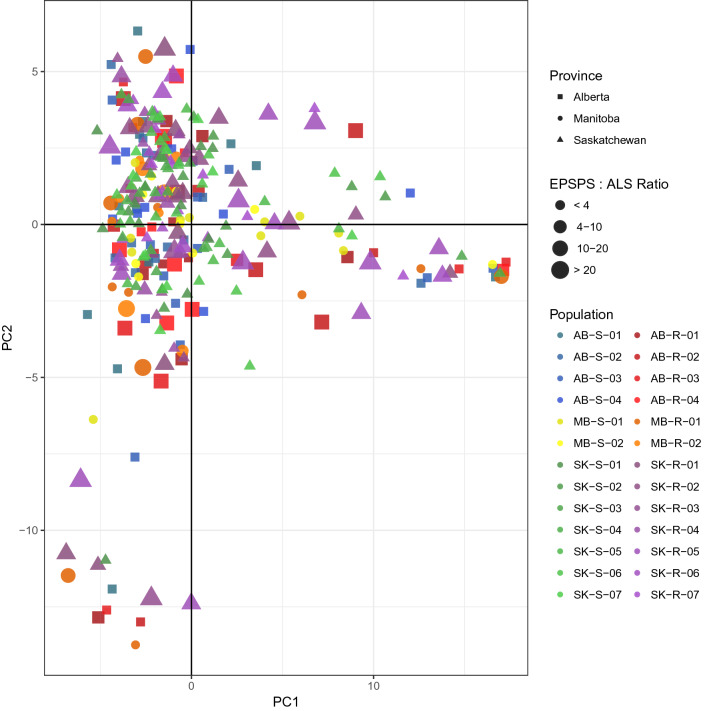
Principal components analysis (PCA) using SNPs from 3248 variable loci with first and second axes account for 23.6% and 11.2% of the variation, respectively, but showing no clustering by EPSPS:ALS ratio (size), population (colour), or province (shape) indicating little to no population structure in this species.

Overall population differentiation (F_ST_) was very low at 0.01, and pair-wise F_ST_ values ranged from 0 to 0.07 (Fig. 1) and did not correlate with geographic distance (Mantel test, r^2^ = − 0.02 p = 0.57). AMOVAs indicated the majority of molecular variation (79.8%) was attributed to individuals, with less than 1% of the variation explained by differences among populations and no variation explained by population status (resistant or susceptible) (Table 2). A principal components analysis (PCA) explained 23.6% and 11.2% of the variance on the first and second axes, respectively, but showed no clustering by province, population or EPSPS type (Fig. 2). Different runs of find.clusters assigned the lowest Bayesian information criterion to different numbers of clusters. However, six clusters were selected with the lowest BIC for both the reference and the de novo loci sets. These groups did not correspond to population, province or EPSPS status (Supplementary Fig. S1), nor did they correspond to clusters or regions within the PCA. When the full sibling groups were included, the optimal number of groups ranged from eight to eleven, but full siblings were not assigned to the same group (Supplementary Fig. S2). The analysis produced by fineRADStructure indicated little population structure with a diffuse pattern of co-ancestry levels averaged by population. Groups of individuals with higher co-ancestry were mixed by province, population and EPSPS status (Supplementary Fig. S3). The overall F_IS_ was calculated as 0.23, but ranged from 0 to 0.42 within populations (Table 1). The proportion of alleles shared by two individuals averaged 75% (range 64–98%), the proportion of polymorphic nucleotide sites (P_n_) averaged 2.73 × 10^–3^ (range 1.02–4.58 × 10^–3^), and the average proportion of loci showing variation within individuals (H_L_) was 13% (range 5% to 23%).

**Table 2:** Analysis of molecular variance: (A) among populations within population type (resistant or susceptible); and (B) among individuals with high or low EPSPS status within resistant populations.

(A)	df	Variance (%)	p-value	(B)	df	Variance (%)	p-value
Between population types	1	− 0.05	0.577	Between populations	11	1.33	0.08
Between populations within type	33	0.79	**0.001**	Between individual EPSPS status within Population	9	− 1.04	0.86
Between individuals within population	260	19.42	** < 0.001**	Between individuals within EPSPS status	109	28.95	** < 0.001**
Within individuals	295	79.83	** < 0.001**	Within individuals	130	75.76	** < 0.001**
Total	589	100.00		Total	259	100.00	

Significance (p-value) was calculated with ade4::randtest.amova based on 1000 permutations and values less that 0.05 are highlighted in bold.

**Figure 3:**
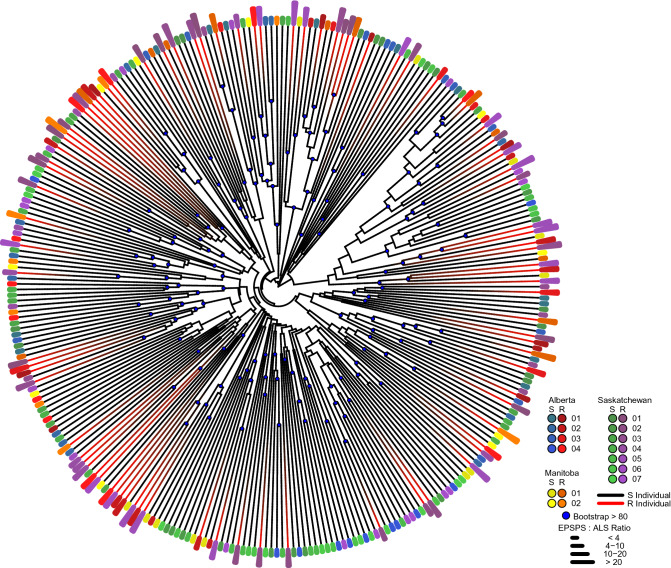
UPGMA tree based on Prevosti’s Genetic distance (see scale at top of tree) for individuals from kochia populations in Alberta, Saskatchewan and Manitoba. Population of origin is represented by the coloured blocks at the tips, while EPSPS:ALS ratio is represented by the size of the bar. Red branches belong to individuals with an EPSPS:ALS ratios of 4 or greater, which are considered resistant to glyphosate, and blue dots indicate nodes with 80 or greater bootstrap support.

The unweighted pair group method with arithmetic mean dendrograms included groupings mixed by province, population and EPSPS status (Fig. 3). While some clustering of individuals with increased EPSPSCN was apparent, for example, a small cluster of high EPSPSCN individuals from Alberta, Saskatchewan and Manitoba populations (e.g. Fig. 3 at 10 o’ clock), others were scattered through the tree likely reflecting the high rates of gene flow rather than multiple independent origins.

The number of migrants among the populations (N_m_) was calculated as 16.4 for comparison to^40^.

### Population statistics and genetic diversity by EPSPS status

AMOVAs indicated that neither population type (Table 2A) nor individual EPSPSCN status within resistant populations (Table 2B) explained genetic variation. Neither the proportion of polymorphic nucleotide sites (P_n_) nor the proportion of variable loci (H_L_) differed by population type or individual EPSPS status (Table 3). Nucleotide diversity estimates were 0.0034 and 0.0035 for individuals with low and high EPSPSCN, respectively. BayseScan indicated that no loci showed evidence of selection when coded by individual or population’s EPSPS status.

**Table 3:** Population statistics comparing low and high EPSPSCN individuals and populations: average proportion of polymorphic nucleotide sites within individuals (10^−3^, P_n_), average proportion of variable loci within individuals (H_L_), and bootstrapped inbreeding coefficients (F_IS_) with confidence interval and p-values.

EPSPS copy number	P_n_	H_L_	F_IS_
**Individuals**			
Low	2.8	0.13	0.227 (0.217–0.237)
High	2.7	0.13	0.279 (0.268–0.290)
p-value	0.11	0.10	0.057
**Populations**			
Low	2.8	0.13	0.230 (0.220–0.240)
High	2.7	0.13	0.256 (0.268–0.290)
p-value	0.33	0.28	0.303

### Alignment to chloroplast

Thirty-six de novo consensus loci aligned to the chloroplast, but all were fixed across individuals. This may indicate that too few regions were used to detect variation, that markers fell in invariant regions, or that there is little variation in the chloroplast. Previous attempts in our laboratory to find variability in *rbc*L, *mat*K, *trn*L-F, *psb*A-H2, *psh*H*-psb*B and *atp-rbc*L yielded only a pair of SNPs across these populations (unpublished data), which may indicate a lack of variation.

## Discussion

Here, we determined that these 26 kochia populations, sampled from across the Canadian Prairie provinces, showed high levels of gene flow. This was indicated by: (1) the very low levels of genetic differentiation (Table 1, Fig. 1); (2) individuals harbouring the majority of genetic variation (Table 2); and (3) the absence of population structure (Figs. 1, 2, 3). This estimate of genetic differentiation for kochia (F_ST_ 0.01) is lower than the moderate level^41^ reported for 13 North Dakota and Minnesota populations based on 45 microsatellites (G_ST_ = 0.09^40^). While we note that calculating the number of migrants from F_ST_ has been criticized as underlying assumptions are likely to be violated^42,43^, our estimate would be six times higher than that of^40^, which suggests a higher level of connection between these populations. The level of genetic differentiation observed here for kochia is also lower than the average genetic differentiation reported for outcrossing wind-pollinated species (G_ST_ value 0.101) based on allozymes^44^. Other weedy species with similarly low F_ST_ include the wind-pollinated grasses *Apera spica-venti* L. (G_ST_ = 0.01, 0.024 for Canadian and European populations, respectively, from allozymes^45^) and *Alopecurus myosuroides* Huds. (F_ST_ = 0.023 from AFLPs^46^), as well as the outcrossing and self-incompatible *Rosa rugosa* Thub. (F_ST_ = 0.045 from microsatellites^47^). Populations of GR plants with similar genetic differentiation include the outcrossing and self-incompatible *Lolium perenne* ssp. *multiflorium* (Lam.) Husnot (F_ST_ = 0.006–0.088 from microsatellites^48^); and some population pairs of the obligate outcrossing species *Amaranthus palmeri* S. Watson in the USA (e.g. F_ST_ = 0.052 for R-S pairs from Arizona from SNPs^49^).

We observed an overall inbreeding coefficient (F_IS_) of 0.23, indicating a 23% higher level of homozygosity than expected by random mating. As material used here was grown from openly pollinated seed, this could indicate that inbreeding is occurring in many populations. Given the low level of genetic differentiation between populations, these high inbreeding coefficients are unusual; plants are generally expected to have either low or high values for both F_ST_ and F_IS_, as self-pollination increases divergence while outcrossing reduces divergence. For example, for two species with low F_ST_ values mentioned above, *R. rugosa* and *A. palmeri*, the F_IS_ values were estimated at 0.043^47^ and 0.016^49^, respectively*.* In contrast*,* central European populations of *Amaranthus retroflexus* L. had inbreeding coefficients similar to the higher values found here at 0.382, but F_ST_ was 0.27 indicating strong population differentiation (allozymes^50^). However, a similar relationship to that observed here was reported for *L. perenne* ssp. *multiflorium,* which had F_IS_ estimates ranging from 0.396 to 0.517 despite low values for genetic differentiation^48^. The authors suggested that this could result from genetic bottlenecks caused by glyphosate selection, but noted that F_IS_ values did not correlate with the frequency of GR plants. Similar processes may be contributing to the high F_IS_ values estimated in this study, since both high and low ESPSCN populations have been subject to selection pressure.

Currently, it is difficult to evaluate kochia’s level of genetic diversity compared to other species, as it is unclear what levels should be expected in weedy or outcrossing plants. This challenge results from the variety of molecular markers used over the last 50 years and the variety of information presented by SNP studies. Genetic diversity (H_E_) in these populations averaged 0.28, lower than the previous report for kochia of 0.35 (Nei’s gene diversity^51^ or *h* in^40^). While this may represent a reduction in genetic diversity, these populations are further north along kochia’s invasion path and may have had lower initial genetic diversity. Alternatively, this difference may be the result of using different genetic markers. Kochia’s genetic diversity is higher than the average of 0.16 for outcrossing wind-pollinated species from allozyme studies^44^. However, the percentage of variable loci (variable vs. variable and fixed loci) was 53%, similar to the 51% of variable allozyme loci in outcrossing species^52^. It is lower than genetic diversity of 0.678–0.824 estimated for GR populations of *L. perenne* ssp. *multiflora*^48^*,* but slightly higher than estimates for 42 GR populations of the highly self-pollinating *Conyza canadensis* (L.) Cronq. (microsatellites; 0.21 (0–0.45)^53^)*.* Unfortunately, few other studies using approaches such as ddRADseq to identify SNPs report the number of loci that were identified, but that were invariant. Our estimate is higher than that for two species of bee-pollinated perennial *Rhododendron* in Japan, where 23% (144 loci of 675) of loci were variable; this was the only other study we located that reported this information^54^. Similarly, kochia’s nucleotide diversity (all sites) was 0.0036, which is lower than estimates of 0.0047 from AFLPS for *A. myosuroides*^46^, but higher than the nucleotide diversity estimated overall for core and invasive populations of *Mercurialis annua* L. (0.0021; SNPs^47^). With increasing numbers of GBS studies examining genetic diversity, we anticipate that sufficient context to evaluate the potential for a particular weed to adapt from standing variation will soon be available^14^.

Whether kochia populations are more or less diverse than expected, there were no differences associated with high EPSPSCN within population or individuals. Similarly, population genetics parameters (e.g. F_ST_, P_n_, H_L_) did not differ between high EPSPSCN populations or individuals and their low EPSPSCN counterparts. The bootstrapped confidence intervals of the inbreeding coefficient for low and high EPSPSCN individuals did not overlap (high EPSPSCN individuals F_IS_ = 0.29, low F_IS_ = 0.24), but random permutation tests indicated no statistical significance (p = 0.057). As a result, if kochia populations are depauperate or rich in comparison to expectations, this is the case whether or not the individuals or populations have high EPSPSCN, indicating that we have no evidence that selection for EPSPSCN has altered genetic diversity in these populations. This study sampled at an early stage in the spread of high EPSPSCN individuals across the Prairie Provinces (i.e. prior to fixation); sampling kochia populations after fixation of GR or after the spread of additional HR genes would provide additional insights.

The high gene flow among populations suggests that kochia on the Canadian Prairies, and perhaps beyond, could be considered a single population. Estimates from tracking tumbling kochia suggest that approximately 90% of seeds are dispersed over the first kilometer^55^, leaving 10%, potentially 3000 seeds^15^, to be dispersed over greater distances. This strong dispersal likely contributes to high connectivity, though human-mediated seed movement is also likely a factor. As a result, beneficial alleles, such as those for herbicide resistance, can be expected to spread rapidly through the species’ range. Further, any selection for a suitable combination of beneficial alleles and genetic background will have all genetic material available in the species to select from when introduced into one area^56,57^. This is congruent with the speed at which ALS mutations spread through the Prairies^27^ and suggests that high EPSPSCN and auxinic resistance are likely to spread as rapidly as ALS resistance. This prediction is supported by Canadian Prairie random surveys showing a rapid increase in incidence of GR (5 to 50% of populations) and auxinic-resistant kochia (0 to 18%) in a 5-year period^25^. With the refinement of our understanding of kochia’s genome and the generation of a chromosome level assembly for the species, it will become possible to use this data to look at the signatures of selection near the EPSPS gene and determine whether this event is associated with a hard or soft sweep and better explore the origin of GR resistance in this species and its consequences^2, 58^.

A potential consequence of this high gene flow is that evolving locally adapted ecotypes would require extremely strong selection in kochia. However, the evolution of locally adapted ecotypes has been considered a key feature of successful invasions^59,60^. Based on this study, we expect the spread of GR will result in little change in kochia’s capacity to evolve additional herbicide resistance from standing variation. Swift, comprehensive, and ongoing action would be needed to curtail the spread of herbicide resistance genes from points of evolution in kochia populations. The species will need to be managed as a whole, as there are no smaller, individually controllable units require coordination and cooperation among producers and levels of government^36^. Future work expanding the geographic coverage of sampling, and investigating the genetic variation of these populations as GR and auxinic resistance spread, would provide further insights.

## Materials and methods

### Plant material

**Figure 4:**
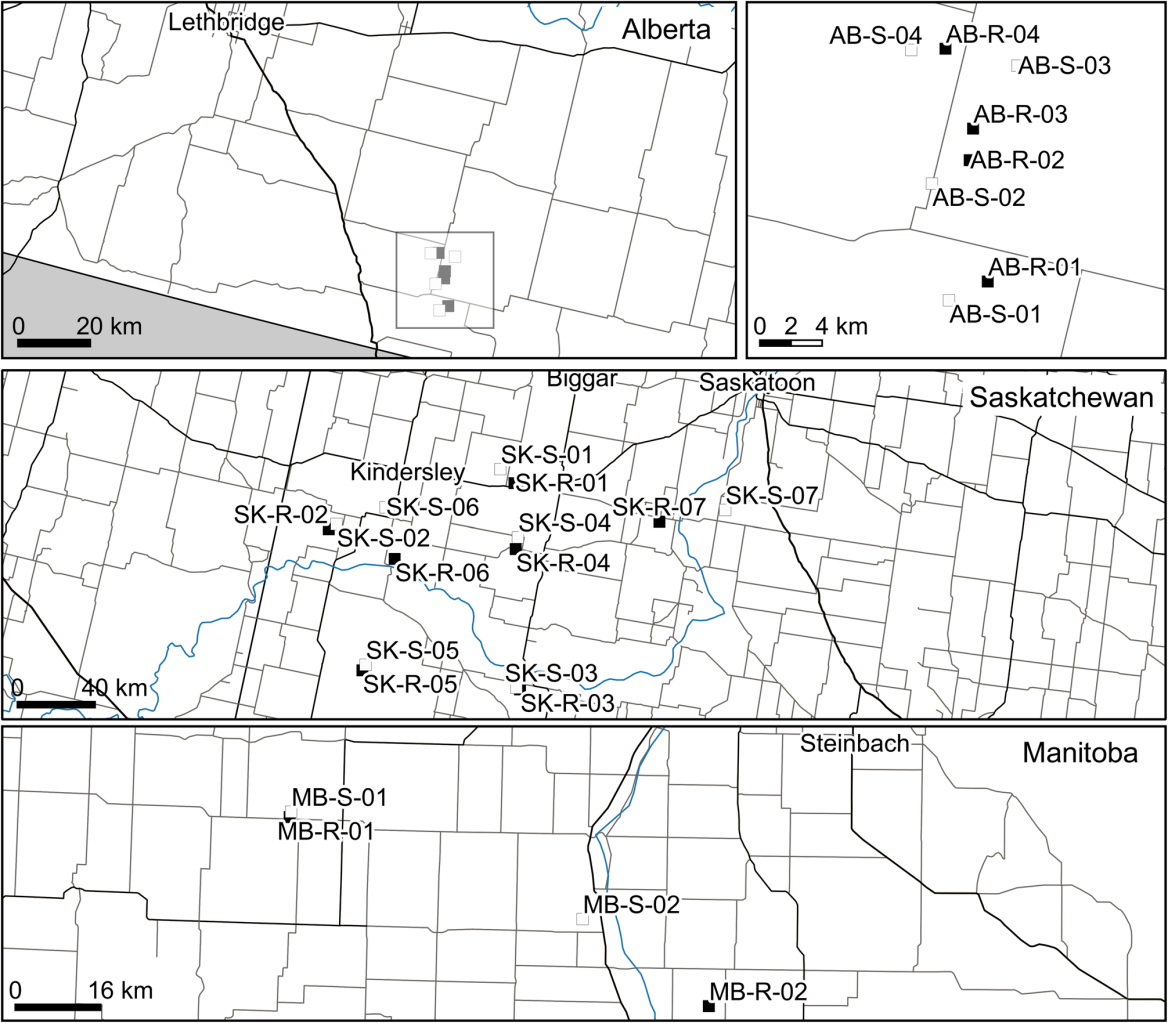
Locations of the kochia populations sampled in Alberta, Saskatchewan and Manitoba, Canada with north toward the top of the figure. Populations where glyphosate resistant individuals were detected in screens by Hall et al.^28^ and Beckie et al.^29^ are filled and contain a “R” in their label while those with no resistance detected in these screens are shown as empty and include an “S” in their labels.

Plant material was from bulk-collected seed from population pairs where high EPSPSCN individuals had been detected (resistant) and from where they had not (susceptible) in relatively close geographic proximity. These populations were sampled and identified during surveys to determine the extent of GR kochia in Alberta (2011, 2012), Saskatchewan (2013) and Manitoba (2013) (Fig. 4)^28–30^. In these surveys, populations were considered resistant if they had individuals not controlled by glyphosate at 900 g ae/ha in greenhouse screens^28–30^. We extracted DNA from twelve individuals from four, seven and two pairs from Alberta, Saskatchewan and Manitoba, respectively. We also used six groups of reciprocally related progeny, sibling plants resulting from reciprocal controlled crosses between high and low EPSPSCN individuals from within populations in Alberta and Saskatchewan^61^. In total, this included 312 individuals from 26 populations and 72 progeny. The maps of the locations of these populations were made in QGIS Desktop 2.18.15^62^ with layers available and downloaded from Natural Earth (https://www.naturalearthdata.com/downloads/).

### DNA sequencing and analysis

#### DNA extraction

Seed were germinated and grown in the greenhouse at Agriculture and Agri-Food Canada’s Ottawa Research and Development Centre. Young leaves were collected and DNA extracted using FastDNA kit (MP BioMedicals, USA). All material from the greenhouses and residual debris following seed cleaning were autoclaved before disposal.

#### Quantitative PCR

Quantitative PCR was used to determine relative EPSPSCN compared to ALS following the method described in^33^. Specifically, two replicates were averaged to determine the EPSPS:ALS ratio. We measured the DNA concentrations of samples using a NanoDrop ND-1000 and 8000 Spectrophotometers (Thermo Scientific, Wilmington, DE, USA), corrected their concentration to 5 ng/μL and conducted Quantitative PCR (qPCR) using an Eppendorf Mastercycler ep cycler. The specific primers for EPSPS: 5′ GGCCAAAAGGGCAATCGTGGAG 3′ and 5′CATTGCCGTTCCCGCGTT TCC 3′^63^, and ALS ALS890F: 5′AGCCTGTGTTGTATGTGGGA 3′ and ALS999R: 5′ AGCGCCCAAACCCATTAAAG 3′^61^ were used and produced products of 102 and 110 bp, respectively. BioRad strip wells containing 10 μL ABI Power Sybr Green MM (2X) (Life Technologies, Hercules, CA, USA), 0.5 μL of the appropriate forward and reverse primer (5 μM), 10 ng of gDNA, and 7 μL of dH2O were used for the qPCR reactions^33^. Cycle parameters were initial denaturing at 95 °C for 15 min, followed by 95 °C for 30 s and annealing and extension at 60 °C for 60 s, for a total of 40 cycles. The ALS reference gene was used to standardize the EPSPSCN using the equation R = 2^−ΔCTsample−ΔCTcalibrator^ to produce the estimate of the ratio between EPSPS and ALS^33^.

#### Double digested restriction enzyme associated markers

Double digested restriction enzyme associated marker library preparation and sequencing were completed at the University of Georgia using a 3DRAD based protocol. The enzymes used to generate the markers were HindIII (A|AGCTT) and NdeI (C|ATATG), and the project was designed to result in 300 million paired end reads for each of four plates.

### Population genetics

Distances between the populations were calculated from their GPS coordinates using the Geographic Distance Matrix Generator v1.2.3^64^, and the population map was created in QGIS 2.18.25^62^ with data from Natural Earth (https://www.naturalearthdata.com).

Data were analyzed using STACKS v1.44^38^ and custom R (3.4.3 “Kite-Eating Tree”) scripts^65^. STACKS (process_radtags) was used to demultiplex and filter data. In total, 1.6 billion reads were received and 1 billion were retained for an average of 1.5 million reads per individual. Stacks parameters were determined using a subset of samples as recommended^66^ and both the de novo and reference pipelines were followed. The parameters used were (M = n =) 5, (m =) 3, the minimum minor allele frequency allowed was 0.05, the maximum observed heterozygosity allowed was 0.7, and a random SNP was used from each locus. For the reference based pipeline, tags were aligned to kochia’s draft genome^67^ using Bowtie 2 version 2.1.0^39^. Average coverage was 20.2 × for the reference pipeline and 15.8 × for the de novo pipeline. Individuals with less than 10 × coverage were excluded. Additionally, we removed loci with more than four alleles identified within full sibling groups, loci with 3 or fewer individuals represented in a population, and individuals with fewer than 60% of the loci. To identify loci associated with the chloroplast, consensus loci sequences were aligned to an assembly of kochia’s chloroplast^67^.

Population genetics parameters were calculated in R^65^. Observed heterozygosity (H_O_), within-population gene diversity (H_S_), overall gene diversity (H_T_), bootstrapped estimates of the inbreeding coefficient (F_IS_) with confidence intervals, and levels of genetic differentiation among populations (F_ST_) were all calculated by hierfstat^68^. Mantel tests were conducted with ade4^69^. Bootstrapped values for F_ST_, associated p-values and Nei’s genetic distance (D_ST_) were calculated with StAMPP^70^. BayeScan^71^ (version 2.1), used with default parameters except an increased prior of 300, produced estimates of F_ST_ with upper and lower limits for each population. The R packages boa^72^ and coda^73^ were used to assess BayeScan results and model convergence. Allelic richness estimates were generated by PopGenReports^74^. Unweighted pair group method with arithmetic mean trees were calculated using poppr^75^, while AMOVAs were calculated with 1000 permutations by poppr.manova with the “ade4” method^69^. The proportion of shared alleles among groups and k-means clustering were estimated (testing k = 1 to 40) with adegenet^76^. The program fineRADStructure was used to further investigate clustering^77^. A custom R script processed STACKS’ haplotype files containing all SNPs for each loci in order to calculate the proportion of polymorphic nucleotide sites (P_n_) and the percentage of heterozygous loci (H_L_). The function oneway_test from coin^78^ estimated the p-value for comparing low and high EPSPSCN individuals or populations using 100,000 permutations. The proportion of shared alleles was calculated by adegenet^76^. Following^40^, the number of migrants were estimated with N_m_ = 0.25((1 − F_ST_)/F_ST_)^79^.

The packages ape^80^, gdata^81^, pegas^82^, phytools^83^, reshape^84^, Hmisc^85^ and vcfR^86^ were used for data handling and manipulation, while ggplot^87^ and colorspace^88^ were used for plotting.

## Supplementary information

Supplementary Legends.

Supplementary Figure S1.

Supplementary Figure S2.

Supplementary Figure S3.

Supplementary Tables.

## Data Availability

Data for this study will be made available by request to the corresponding author.
