# Supplementary material for: High gene flow maintains genetic diversity following selection for high EPSPS copy number in the weed kochia (Amaranthaceae)
Source: Sci Rep. 2020 Nov 2;10:18864. doi: 10.1038/s41598-020-75345-6 (PMC7608611; doi:10.1038/s41598-020-75345-6)
Supplement: Supplementary file 1 — Supplementary Legends. [file 41598_2020_75345_MOESM1_ESM.docx]

**High gene flow maintains genetic diversity following selection for high EPSPS copy number in the weed kochia (Amaranthaceae)**

Sara L. Martin^1^, Leshawn Benedict^1^, Wei Wei^2^, Connie A. Sauder^1^, Hugh J. Beckie^3^, and Linda M. Hall^4^

1 Agriculture and Agri-Food Canada, Ottawa Research and Development Centre, Ottawa, Ontario, Canada

2 State Key Laboratory of Vegetation and Environmental Change, Institute of Botany, Chinese Academy of Sciences, Beijing, China

3 Australian Herbicide Resistance Initiative, School of Agriculture and Environment, University of Western Australia, Perth, Australia

4 Agricultural Food and Nutritional Science, University of Alberta, Edmonton, Alberta, Canada

Supplementary Fig. 1. K-means clustering and DAPC analyses identified eight groups as being a potential best fit for the data when sibling groups derived from six of the populations were included. However, these full sibling sets were not allocated to the same group (S = susceptible with ≤4 EPSSP:ALS ratio) or R = resistant with >4 EPSSP:ALS ratio).

Supplementary Fig. 2. K-means clustering and DAPC analyses identified six groups as being a potential best fit for this data. However, these groupings did not correspond to population, province or EPSPS status (S = susceptible with ≤4 EPSSP:ALS ratio) or R = resistant with >4 EPSSP:ALS ratio).

Supplementary Fig. 3. Degree of co-ancestry between individuals (above the diagonal) and averaged by population (below diagonal) with lower co-ancestry levels in yellow darken to red with increased co-ancestry as estimated by fineRADstructure. The expectation would be that population structure would be visualized by darker blocks clustered along the diagonal line indicating groups of individuals with greater levels of genetic similarity compared to individuals not in that group (population). Here the levels of genetic similarity are largely homogenous with some individuals from different populations/provinces showing increased genetic similarity due to gene flow.
