# Supplementary figures and images for: High gene flow maintains genetic diversity following selection for high EPSPS copy number in the weed kochia (Amaranthaceae)
Source: Sci Rep. 2020 Nov 2;10:18864. doi: 10.1038/s41598-020-75345-6 (PMC7608611; doi:10.1038/s41598-020-75345-6)

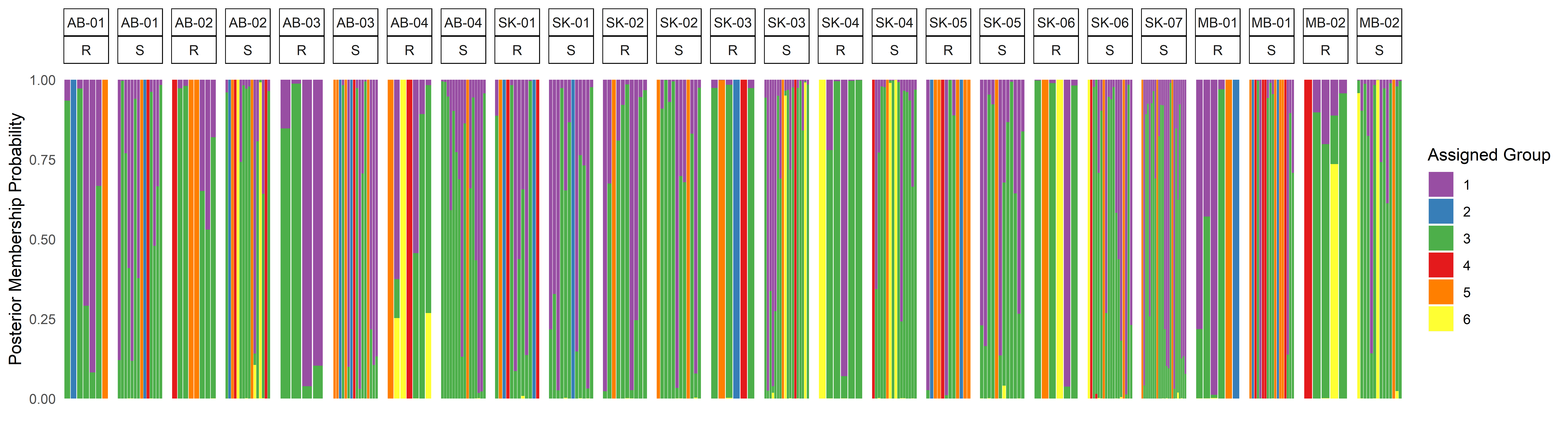

Supplement: Supplementary file 2 — Supplementary Figure S1. [file 41598_2020_75345_MOESM2_ESM.tiff]

|       |       |       |       |       |       |       |       |       |       |       |       |
|-------|-------|-------|-------|-------|-------|-------|-------|-------|-------|-------|-------|
| AB-01 | AB-01 | AB-02 | AB-02 | AB-03 | AB-03 | AB-04 | AB-04 | SK-01 | SK-01 | SK-02 | SK-02 |
| R     | S     | R     | S     | R     | S     | R     | S     | R     | S     | R     | S     |

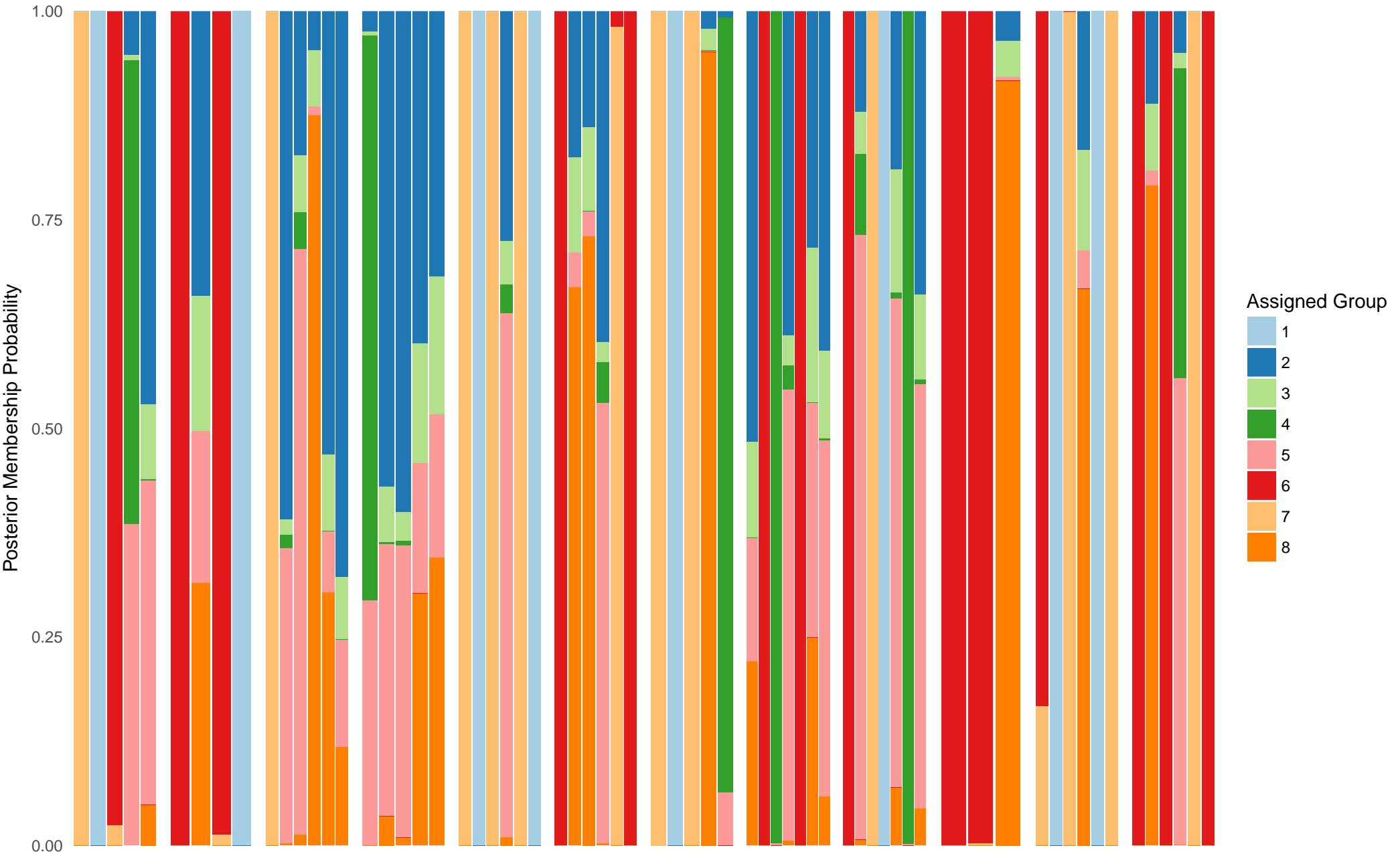

Supplement: Supplementary file 3 — Supplementary Figure S2. [file 41598_2020_75345_MOESM3_ESM.pdf]

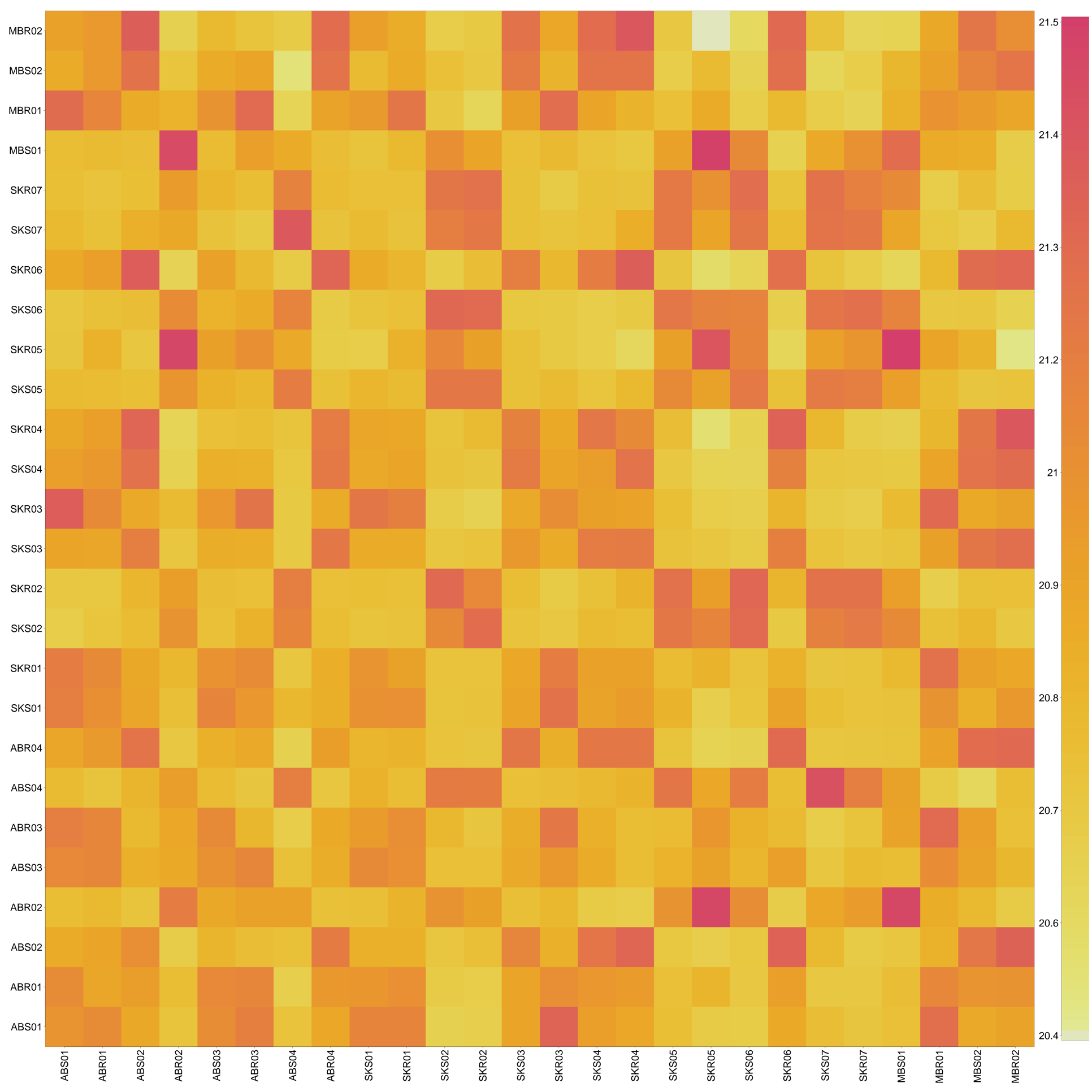

Supplement: Supplementary file 4 — Supplementary Figure S3. [file 41598_2020_75345_MOESM4_ESM.pdf]
